# Programmable acoustic modular microrobots

**DOI:** 10.1007/s12213-024-00175-y

**Published:** 2024-08-03

**Authors:** Subrahmanyam Cherukumilli, Fatma Ceren Kirmizitas, David P. Rivas, Max Sokolich, M. Cagatay Karakan, Alice E. White, Sambeeta Das

**Affiliations:** 1 Department of Mechanical Engineering, University of Delaware, Newark, 19711 DE USA; 2 Department of Animal and Food Sciences, University of Delaware, Newark, 19711 DE USA; 3 Department of Biomedical Engineering, Boston University, Boston, 02215 MA USA; 4 Department of Mechanical Engineering, and the Departments of Biomedical Engineering and Materials Science and Engineering, Boston University, Boston, 02215 MA USA

**Keywords:** Modular microrobots, Programmable microstructure, Magneto-acoustic/hybrid microrobot, Cell manipulation, Cell patterning

## Abstract

The field of microrobotics has emerged as a promising area of research with significant applications in biomedicine, both in vitro and in vivo, such as targeted cargo delivery, microsurgery, and cellular manipulation. Microrobots actuated with multiple modalities have the potential for greater adaptability, robustness, and capability to perform various tasks. Modular units that can reconfigure into various shapes, create structures that may be difficult to fabricate as one whole unit, and be assembled on-site, could provide more versatility by assembly and disassembly of units on demand. Such multi-modal modular microrobots have the potential to address challenging applications. Here, we present a biocompatible cylindrical microrobot with a dome-shaped cavity. The microrobot is actuated by both magnetic and acoustic fields and forms modular microstructures of various shapes. We demonstrate the use of these microrobots for cellular manipulation by creating patterns on a surface.

**Supplementary Information:**

The online version contains supplementary material available at 10.1007/s12213-024-00175-y.

## Introduction

Microrobots are micro-scale robots that can be used for a variety of applications, especially in the field of bioengineering, due to their small size [1]. Microrobots can be actuated by magnetic, acoustic, light, and chemical techniques [2, 3]. Out of these, magnetic and acoustic actuation mechanisms are well suited for clinical applications due to their strong penetration, biocompatibility, and minimal invasiveness [4]. In addition, the integration of two or more actuation methods can provide better control and navigation [5]. For example, the integration of magnetic and acoustic actuation in a single microrobot has been used for a variety of applications such as biomedical [6], shape morphing [7], and cell targeting [8]. By implementing multi-actuation methods, researchers can enhance the microrobot’s functionality and adaptability [9], particularly in scenarios where a combination of actuation methods is required to navigate complex environments or achieve intricate tasks.

Researchers have employed various fabrication techniques, including micro-scale 3D printing, to develop microrobot designs for many applications [10–12]. Although they demonstrated better capabilities in performing specific tasks, limitations still exist, including a lack of versatility while navigating non-homogeneous environments or completing tasks in vivo [13].

At the macro-scale, modular robotics has gained extensive research interest for various applications due to modular robots have their versatility, scalability, robustness, and adaptability [14]. At the micron scale, the formation and reconfiguration of modular units show better potential to perform tasks more efficiently, especially in biomedical applications, and to overcome fabrication limitations. Modular structures have been made from nanoparticles and micro-scale building blocks [15, 16]. Researchers have also demonstrated the assembly and disassembly of microswimmer shapes into different configurations using helical [17] and spherical units [13] that can be driven using magnetic actuation. In addition, the feasibility of employing modular units for cellular manipulation has been shown in Ref. [18].

One of the prominent applications where microrobots are effective is single-cell manipulation. Traditionally, single-cell manipulation has been done by micromanipulators which have a bigger and more complex experimental setup that also requires trained scientists to perform the experiments. These manipulation systems can be micropipettes [19], dielectrophoretic trapping [20], optical tweezers [21] or acoustofluidic devices [22]. Microrobot-facilitated single-cell manipulation could improve efficiency and control compared to conventional methods [23–25] and can be employed in cell patterning, microsurgery, and tissue engineering. Conventional cell patterning methods such as microcontact printing [26], cell trapping [27], stamping [28], and microfluidic-based [29] have been developed to recapitulate the single-cell level interactions. Although these methods are effective, there are still challenges to overcome. The complexity of the external control setup, biocompatibility, and unattached cell waste during the process are major concerns [30, 31]. Microrobot-guided cell patterning meets the requirements of ease of operation, low cost, and biocompatibility, so several different magnetic microrobots have been employed for cell patterning. A distinct advantage of microrobots over micromanipulators is that they are untethered and hence are capable of being employed in closed environments such as microfluidic chips or hard-to-access regions within the body [6]. Thus, single-cell manipulation using microrobots shows high accuracy, maneuverability, and controllability [32, 33]. By utilizing the microrobots, a single cell can be precisely cached, carried, and released at the target site [34–36].

In this paper, we present a novel microrobot design that is capable of making programmable modular units. A layer of nickel coating on the semi-lateral surface of the individual units allows for magnetic actuation and magnetic dipole-dipole attraction-based assembly of the modular units. A cavity within the microrobots traps a bubble, which also enables acoustic propulsion. Using these mechanisms, we show the transport of mammalian cells from one location to another and form specific patterns using individual microrobots.

**Fig. 1:**
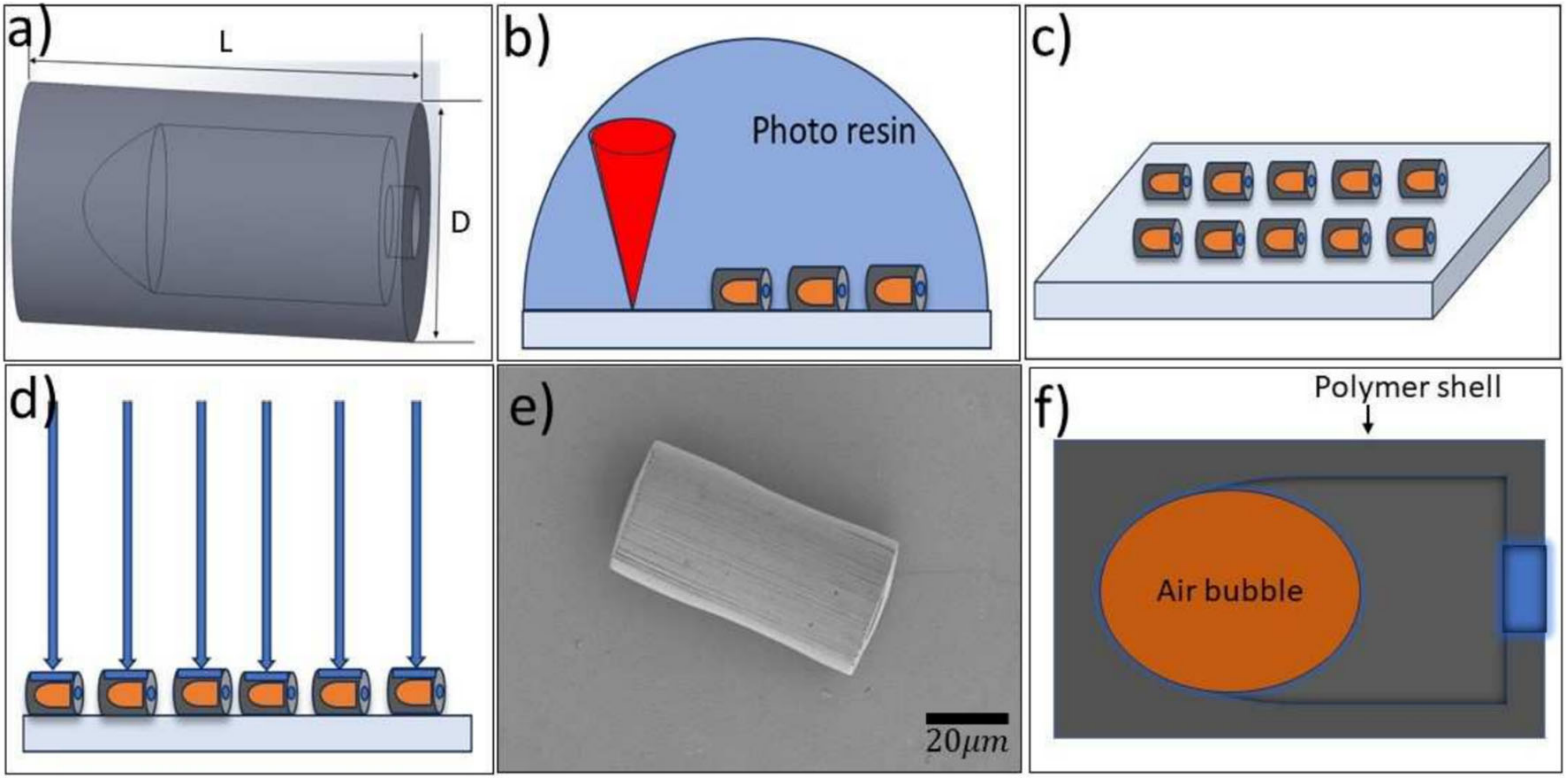
Design and Fabrication process. a) Overview of the microrobot design of length L (80 $\mu$m and 40 $\mu$m ) and diameter D (40 $\mu$m and 20 $\mu$m), respectively. b) 3D printing microrobots on a silicon chip using a two-photon direct laser writing technique. c) Overview of the printed microrobots on the silicon chip after removing the excess resin. d) Illustration of the nickel vapor deposition on the silicon chip. e) The scanning electron microscope image of the nickel-layer deposited microrobot. f) The sectional view of the microrobot with the air bubble in the deionized (DI) water suspension

**Fig. 2:**
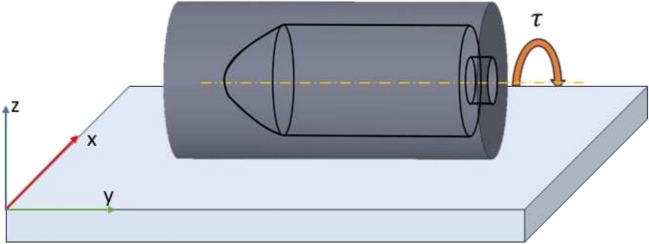
Illustration of the rolling motion of the microrobot

## Methods

### Design and fabrication

The microrobot design was inspired by the work in Ref. [6] and we developed it as illustrated in Fig. 1. The microrobot is cylindrical and contains a dome-shaped cavity. The design of the cavity allows it to retain an air bubble once immersed in water. Two sizes were made, one of 40 $\mu$m length and 20 $\mu$m diameter and another of 80 $\mu$m length and 40$\mu$m diameter. The microrobots were fabricated via two-photon direct laser writing lithography technique, using Nanoscribe Photonic Professional GT+ equipped with a 63$\times$ objective (NA=1.4). Using IP-Dip2 as the photoresist, microrobots were printed on diced silicon chips as 20$\times$20 arrays (see Fig. 1b). Subsequently, unpolymerized resist was rinsed from the chip and the microrobots by placing the chips vertically into a propylene glycol monomethyl ether acetate bath for at least 1 hour, followed by an isopropanol bath. The remaining solvent was cleaned from the microrobots by dipping the chip into a NOVEC 7100 (3M) bath for a minute and slowly removing it (see Fig. 1c). Later, the microrobots are coated with a 100 nm nickel layer using the Dual vapor E-BEAM deposition technique. The deposition is perpendicular to the curved surface, making the cylinders half-coated with the nickel (see Fig. 1d). This nickel coating enables the microrobots to be controlled by an external magnetic field.

### Actuation

The actuation of the microrobot is achieved using both magnetic and acoustic fields. This section discusses the two types of actuation mechanisms employed in this work.

#### Magnetic actuation principle

Magnetic fields are generated by electromagnets that produce uniform fields in a specified direction. We use a custom-built 3D Helmholtz coil system that is controlled by an Xbox joystick to produce uniform and rotating magnetic fields. The fields generate a magnetic torque on the microrobot, which is given by

1 $$\begin{aligned} \varvec{\tau } = \varvec{\mu } \times \varvec{B}, \end{aligned}$$

where $\mu$ is the Magnetic moment, and B is the Magnetic Field. This torque results in the rolling motion of the microrobot when subjected to a rotating magnetic field Fig. 2. For example, a magnetic field rotating in the *xz* plane results in a rolling motion in the negative or positive *x* direction, depending on the sign of the rotating field. To further understand the magnetic effect on the microrobot, we began with a zero magnetic field and then applied an 8 mT magnetic field along the *z*-component. The microrobot’s orientation did not change while lying on the glass slide’s surface along its major axis.

The rotating magnetic field can generally be expressed in terms of its components along each axis,

2 $$\begin{aligned} {B_x} = -B[\cos (\gamma )cos(\alpha )\cos (\omega t) + \sin (\alpha )\sin (\omega t)] \end{aligned}$$

3 $$\begin{aligned} {B_y} = -B[\cos (\gamma )\sin (\alpha )\cos (\omega t) - (\cos (\alpha )\sin (\omega t))] \end{aligned}$$

4 $$\begin{aligned} {B_z} = B\sin (\gamma )\cos (\omega t), \end{aligned}$$

where $\gamma$ is the polar angle from the *z* axis, $\alpha$ is the azimuthal angle from the *x* axis, *B* is the magnetic field magnitude, and $\omega$ is the frequency of the field. The default $\gamma$ is 90$^\circ$, and changing $\alpha$ allows us to steer the microrobots when actuating under magnetic control.

#### Acoustic actuation principle

**Fig. 3:**
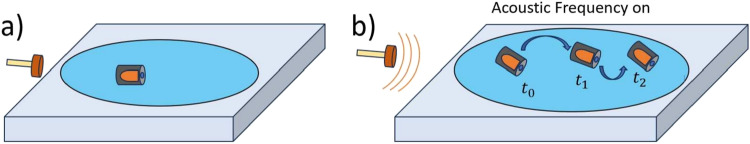
Acoustic Schematic. a) The initial position of the microrobot. b) Movement of the microrobot over time by applying acoustic frequency

Acoustic waves are generated by piezo-electric transducers. The transducers generate waves at various frequencies that result in the movement of microrobots. The actuation of the microrobot is controlled by varying the transducer frequency. Figure 3 shows an illustration of the acoustic movement of the microrobots.

Acoustic-actuated microrobots can be propelled by the oscillations of a trapped bubble within the microrobot, which is most intense when the acoustic field frequency reaches the resonance frequency of the bubble [6]. More details of the acoustic propulsion mechanism are provided in the Results section.

### Control system

An open loop control system is implemented for the magnetic control of the microrobots. The 3D Helmholtz coil system is capable of generating a uniform magnetic field that can perform the rolling and steering of the microrobots. The control system mainly consists of a Raspberry Pi Model 3 for computational capability, H-Bridge PWM Drivers for varying and controlling the direction of the current flow in the coils, and a DDS Module to digitally generate the waveforms that are transmitted to the piezoelectric transducer resulting in the formation of the acoustic waves. An external power system is employed to input the required current to the system. The real-time control of the current to each electromagnetic coil is achieved by using a custom graphical user interface developed in Python using the Tkinter and Gpiozero libraries. For more details on how the system is developed, see Ref. [37].

### Cell culture

Chinese Hamster Ovary (CHO) cells were cultivated in Dulbecco’s Modified Essential Medium/Nutrient Mixture F-12 (DMEM/F-12, Gibco, BenchStable, USA) supplemented with 10% Fetal Bovine Serum (F7524, Sigma, USA) and 1% penicillin-streptomycin and maintained at the physiological conditions (37 $^\circ$C with an atmosphere of 5% CO2). Cells were grown regularly until reaching approximately 90% confluency, and subsequently subcultivated with TrypLE™(12604-013, GIBCO, USA). Grown and proliferated cells were either dissociated and replated, or used in the experiments until the eighth passage under the standard cell culture conditions.

### Cell viability assays

Quantitative cell viability was assessed via trypan blue exclusion assay, a 10 $\mu$l of trypsinized and resuspended cell suspension was mixed with 10 $\mu$l of 0.4% trypan blue solution. Cells were directly counted using a cell counter (Nexcelom Cellometer Vision Trio Cell Profiler, USA), and morphology was determined using a light microscope (ZOE Fluorescent Cell Imager, USA) in three replicates. Qualitative cell viability was determined by a commercially available LIVE/DEAD Viability/Cytotoxicity Kit for mammalian cells (Invitrogen, L3224) by following the manufacturer’s instructions. Cell viability was tested to determine the toxicity of the external magnetic field itself at a cellular level by comparing the three different groups; cells with and without microrobots, and control cells without microrobots and not exposed to any magnetic field. The external magnetic field was static at 4 mT, and treatment was for 5 minutes. The kit has two different fluorescent dyes that work to detect cytoplasmic activity and membrane integrity. Calcein AM stains viable cells after passing the cell membrane and being hydrolyzed by cytoplasmic enzymes, and ethidium homodimer-1 stains the dead cells by binding the DNA after passing the damaged cell membrane. Calcein AM induces the emission of green fluorescence light at 517 nm whereas ethidium homodimer-1 induces the emission of red fluorescence light at 617 nm. Cells were incubated with the microrobots after magnetic actuation, and seeded into a 6-well plate (Costar, Corning, USA) as 1x105 cells/ml per well. After overnight incubation, the cells were stained with 0.1% calcein AM and 0.2% ethidium homodimer-1 and incubated at the standard culture conditions for 25 min. The samples were washed with phosphate-buffered saline and imaged under a fluorescent microscope. Each analysis was performed three times under the same conditions.

### Experimental setup

**Fig. 4:**
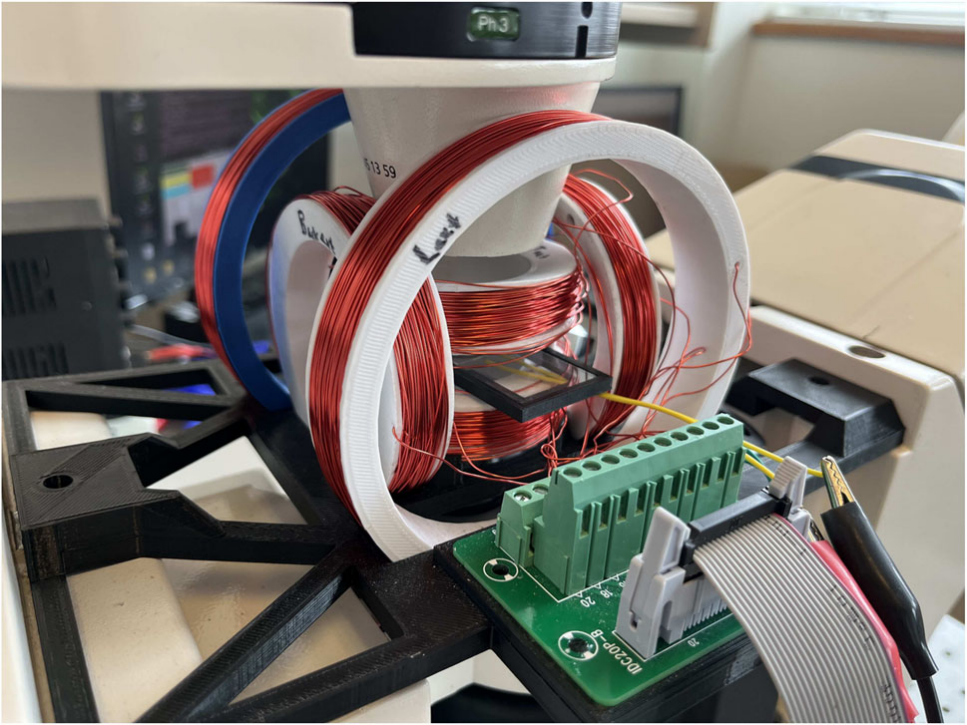
Experimental setup

The experimental setup consists of the 3D Helmholtz coil system, a DDS wave generator, and the control system mentioned above. The 3D Helmholtz coil system is placed on an inverted Axiom 200 Microscope and generates a uniform magnetic field at the center Fig. 4. The microrobots are suspended in DI water and pipetted onto a glass slide. The transducer is attached to the glass slide with an optical adhesive.

## Results

### Acoustic actuation

Microrobots were collected from a silicon wafer in a small drop of DI water which was placed on a glass slide. The microrobots are buoyant due to the enclosed bubble and therefore require a glass coverslip to position them on the glass surface. Individual microrobots were acoustically actuated (see Video S3) by applying a frequency near the resonant frequency of the bubble, which we found to be around 114 kHz for 40 $\mu$m diameter microrobots and 50 kHz for 80 $\mu$m diameter microrobots.

Previous reports on smaller, 3-5 $\mu$m, acoustic microrobots have shown that this actuation mechanism is dominated by an acoustic streaming propulsive force, which is due to fluid flow produced by the oscillating bubble [6, 38, 39]. The bubble is also subject to acoustic radiation forces, known as Bjerknes forces, caused by a pressure gradient in the acoustic field [6, 40, 41]. There are two types of Bjerknes forces, primary and secondary. Primary Bjerknes forces are caused by an exterior acoustic field, while secondary Bjerknes forces are due to acoustic waves scattering off of other bubbles. For example, a bubble can move to the nodes of a standing wave due to the primary Bjerknes force, while it can be attracted to other bubbles due to the secondary Bjerknes force. In the case of a bubble oscillating near a rigid boundary, the zero velocity boundary condition is satisfied by considering an image bubble oscillating in phase with the real bubble, hence resulting in an attraction to the surface via the secondary Bjerknes force [6]. Therefore, the bubble microswimmers are acted upon by a downward secondary Bjerknes force that also results in a torque that tends to orient the microrobots in an upright position with the open side facing the surface [6, 39]. We also sometimes observe this behavior (see Video S3), although often the microrobots instead lay flat with their long axis parallel to the surface, probably due to the strong gravitational torques on these much larger microrobots or their more elongated structure. Microrobots that do stand upright can be moved by magnetically tilting them in a particular direction, in accordance with previous reports on smaller microrobots [6, 39].

Horizontal forces on the microrobots are due to the primary Bjerknes force and acoustic streaming-induced forces. One can show that the ratio of the acoustic streaming force, $F_{S}$, to the primary Bjerknes force, $F_{PB}$ is given by, $\frac{F_{S}}{F_{PB}} = \frac{\epsilon \rho _l c_l f r}{P_{ac}}$, where $\epsilon$ is the fractional amplitude of the oscillation of the bubble radius, $\rho _l$ is the mass density of the liquid, $c_l$ is the speed of sound in the liquid, *f* is the acoustic frequency, *r* is the bubble radius, and $P_{ac}$ is the pressure amplitude of the acoustic waves [38]. In the case of smaller acoustic microswimmers reported previously, this ratio has been estimated to be large ($\sim$10), therefore acoustic streaming was determined to be the dominant propulsion mechanism [6, 38, 39]. Our microrobots contain bubbles that are $\sim$10 times larger, but the resonant frequency is about 20 times smaller. The value of $\epsilon$ is likely similar to that of the smaller bubbles of Refs. 6, 39 and therefore we take it to be $10^{-2}-10^{-3}$ [39]. The other values are constants, except the acoustic pressure, $P_{ac}$, which we expect is roughly equal to $10^4$ Pa [6]. Therefore, the ratio of acoustic steaming forces to primary Bjerknes forces is 3-30 in our case. Hence we expect that primary Bjerknes forces are of less importance than acoustic streaming forces. This is supported by our observation of flow vortices produced near the microrobot’s open end, which we visualized with tracer particles (see Video S2), in accordance with the flow expected due to acoustic streaming [41]. Additionally, the observation that microrobots that stand upright once the acoustic field is turned on only move rapidly once magnetically tilted (hence exhibiting the same behavior as previous reports), indicates that acoustic streaming is the driving mechanism in these cases as well. In these cases, the tilt of the microrobot creates a horizontal component of the streaming force which moves the microrobot parallel to the surface.

Occasionally, microrobots that were oriented parallel to the surface were observed to move backward, i.e. in the direction of their open end. We do not believe this was due to primary Bjerknes forces since a bubble experiencing an acoustic gradient force would move towards a node of the standing wave and, due to the extremely long wavelengths (15-30 mm) of the standing sound waves at the resonant frequencies, this would be expected to result in motion in only one direction rather than always towards the open end of the microrobot. Although we are unsure of the mechanism responsible for the backward motion, we speculate that it could be due to a slight deformation of the shapes of these microrobots leading to complex flows around the microrobots.

The bubble-resonant frequencies of our microrobots are about 50 and 114 kHz (for 80 $\mu$m and 40 $\mu$m long microrobots, respectively). At particular frequencies above 1 MHz and at sufficiently large amplitude acoustic pressures, we observe that passive tracer particles gather in certain regions (see Video S2), similar to the observed effect of sand particles on Chladni plates. The driving mechanism, in this case, is due to acoustic radiation forces that push the tracer particles toward low-pressure nodes of the acoustic field. In the example video shown, the separation between the regions of high particle densities is about 700 $\mu$m. This is similar to the distance one would expect based on a simple 2D lattice of nodes and anti-nodes, where the separation is approximately 0.7 times the wavelength of the standing wave [42]. The wavelength is equal to $c_l/f$, which is about 1000 $\mu$m in the case of a 1.4 MHz acoustic wave and a speed of sound in the water of 1500 m/s. Therefore the distance between the nodes would be about 700 $\mu$m, which is similar to the measured value. We also measured the distance between the particle clusters at a frequency of 3.3 MHz and found the distance to be about 270 $\mu$m, which is also similar to 0.7 times the wavelength (320 $\mu$m). We note, however, that the shape of the nodal patterns could be more complex than a simple lattice structure, although the inverse relationship between wavelength and nodal spacing seems to agree well with our findings.

Although bubble lengths were observed to vary somewhat over time from microrobot to microrobot, the resonant frequency was not noticeably altered. This observation is consonant with theoretical work that predicted a very weak dependence of the resonant frequency on the aspect ratio of elongated bubbles [43].

Microrobots at the air-liquid interface could be moved by acoustic fields at frequencies above 1 MHz and gathered in clusters, presumably at the low-pressure nodes of the acoustic field, as discussed in the next section.

**Fig. 5:**
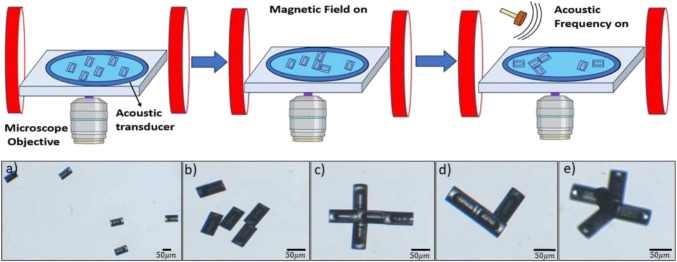
Formation of the modular units. (Top): Schematic of the experiments (Bottom): Microscope images of the 80 $\mu$m microrobots taken at 10$\times$ and 5$\times$ magnification - a) Initial state of microrobots after placing on a glass slide. b) Microrobots under the magnetic field. c,d,e,) Various shapes were programmed during the experiments

### Modular units

Here we demonstrated the ability of the modular microrobots to form specific shapes. We have done this at the glass surface and the air-water interface. To do this, microrobots suspended in DI water were pipetted onto a glass slide. Due to the buoyancy of the bubble inside the microrobot, they remain at the air-water interface. A coverslip was placed on top of the droplet to get them to reside on the glass surface. First, we will discuss modular formation at the interface.

To manipulate the position of the microrobot, a uniform magnetic field is applied along the *x* and *y* directions using the joystick which creates microrobot motion, possibly due to a combination of buoyancy, surface tension, and induced curvature of the interface. Stronger fields produced faster motion of the microrobots. Once in proximity, the microrobots attract and attach due to the dipole-dipole interactions between the Ni-coated microrobots. Their magnetic polarity results in a preferred orientation of each microrobot with the others, typically parallel or perpendicular to one another. By adjusting the magnitude of the current to the coil system and the application of time-varying magnetic fields, varying shapes are formed. The formation of the shapes is affected by the number of microrobots, their proximity to each other, the amount of magnetic field applied by varying the current to the coil system, and the thickness of the Ni coating layer. If the microrobots are too far the shape formation is not achievable as the magnetic attractions between the microrobots are not strong enough to join together.

Modular components could be assembled at the interface by applying acoustic waves (see Video S4) which could increase the number and speed of assembly. We attribute this to the formation of acoustic nodes at the interface, gathering the individual components together in the low-pressure regions. The modular units remain intact once the acoustic field is removed due to the magnetic dipole attraction between them. The typical frequency applied to do this was between 1-1.4 MHz at 6-20 Volts. As mentioned earlier, the distance between nodes at this frequency is expected to be about 700 $\mu$m. This is small enough to allow for a well-localized grouping of the modular units. The modular microrobots could be rotated using magnetic fields, although this did not result in a noticeable change in the propagation direction of the microrobots, therefore indicating that acoustic radiation forces are the primary driving mechanism. The microrobot’s direction of motion could be adjusted by changing the acoustic frequency.

Modular assemblies could also be formed at the glass surface after a coverslip was placed atop the liquid droplet to force the microrobots to reside on the surface. Then, rotating magnetic fields were used to roll the microrobots near each other. The formation was not as efficient or stable at the surface compared to the interface due to the additional friction with the substrate inhibiting their magnetic attraction-based aggregation.

**Fig. 6:**
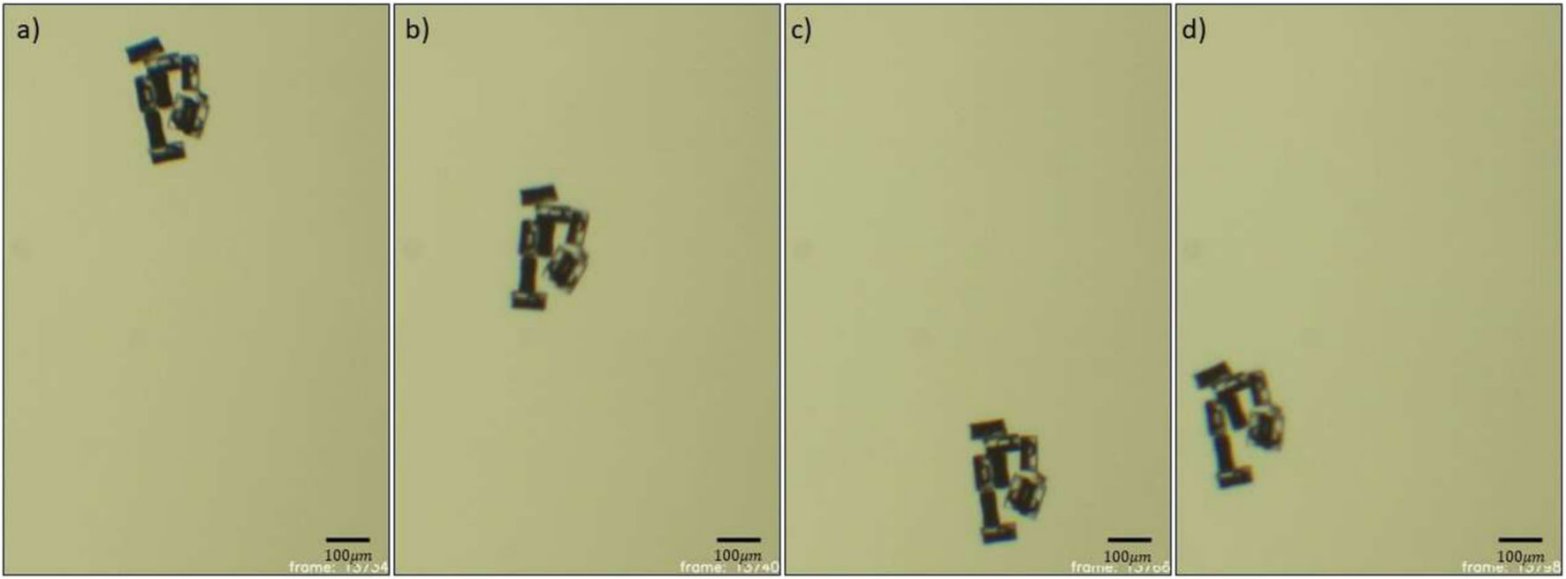
Actuation of the modular units. a,b,c,d) Frames showing the acoustic actuation of the modular microrobot

Collections of three to five units resulted in characteristic shapes such as ‘L’, ‘T’, and ‘X’ (see Fig. 5c-e)). The shape of the modular units formed was stable under acoustic actuation, although applying magnetic fields in different directions can result in a change in the modular structure, as noted above. The modular units formed can also be oriented in different directions by applying rotating magnetic fields. Actuation of the modular units was achieved by applying acoustic fields at the resonating frequency of the bubbles as in Fig. 6.

For modular microrobots on the glass surface, movement was attained at the bubble resonant frequency (see Video S4). The modular microrobots that were actuated at the resonant frequency could also be steered in any direction using applied magnetic fields (see Video S4).

**Fig. 7:**
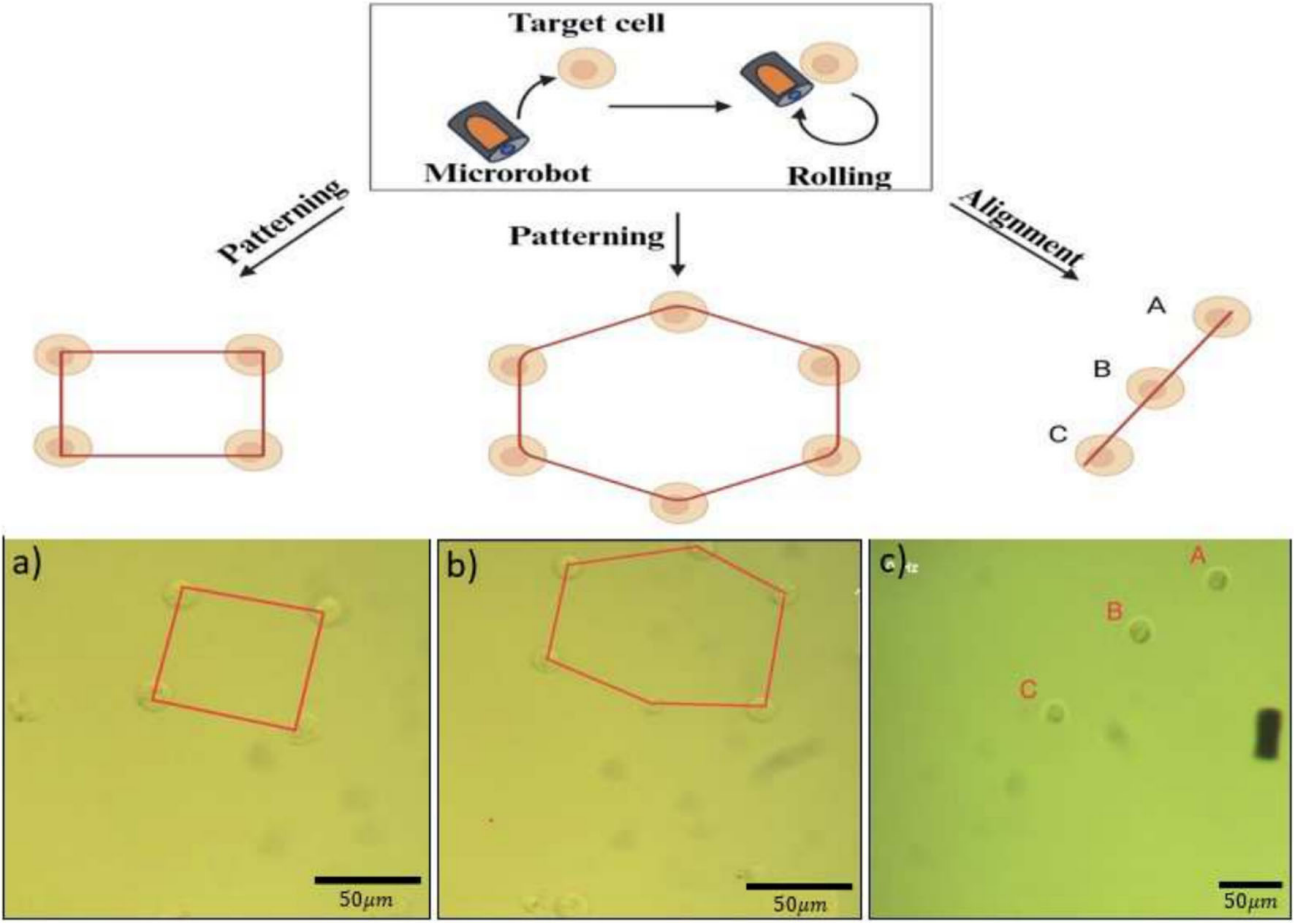
Schematic of single CHO cell arrangement in specific patterns using cylindrical microrobots. a, b) Square and hexagon patterns, c) Aligned CHO cells via microrobots

### Cell patterning

The potential use of our microrobots for cellular manipulation and patterning was investigated. To meet the requirements of cell delivery, the microrobot design and structure are very crucial in terms of maneuverability and precise control. In terms of cellular manipulation, caging and pushing/pulling are considered the most forthright manners, and magnetic field-driven microrobots are one of the most effective carriers in the system [44]. At a magnetic field of 10 Hz and 4 mT, the microrobots could roll with a velocity of 33 $\mu$m/s in cell culture media, which is considered suitable for single-cell manipulation. Figure 7 and Video S4 show CHO cell arrangement by a single microrobot in a straight line and hexagon patterns via rolling motion. The microrobot approaches, picks up, and releases a single cell to create specific geometric patterns. Once the microrobot comes into proximity to a CHO cell, Van der Waals force between the two surfaces can lead to an attachment [45]. It should be noted that this attachment is crucial because it allows the microrobot to effectively transport cells without the need for a chemical treatment or needle system while maintaining biocompatibility. The straight line comprises 3 CHO cells, the hexagon comprises 6 CHO cells, firstly 4 cells to create a square, then an additional 2 more CHO cells to create a hexagon pattern. The duration of patterns is 90 seconds for straight lines and 52 seconds for square and hexagon shapes. The results demonstrated the maneuverability and controllability of the microrobots for single-cell manipulation. In the literature, similar cellular manipulation capabilities of these microrobots have been shown to create a matchstick man pattern with NIH/3T3 cell aggregates [46], “T” and “U” shapes with CHO cells [35], and “HIT” pattern with NIH/3T3 [32].

**Fig. 8:**
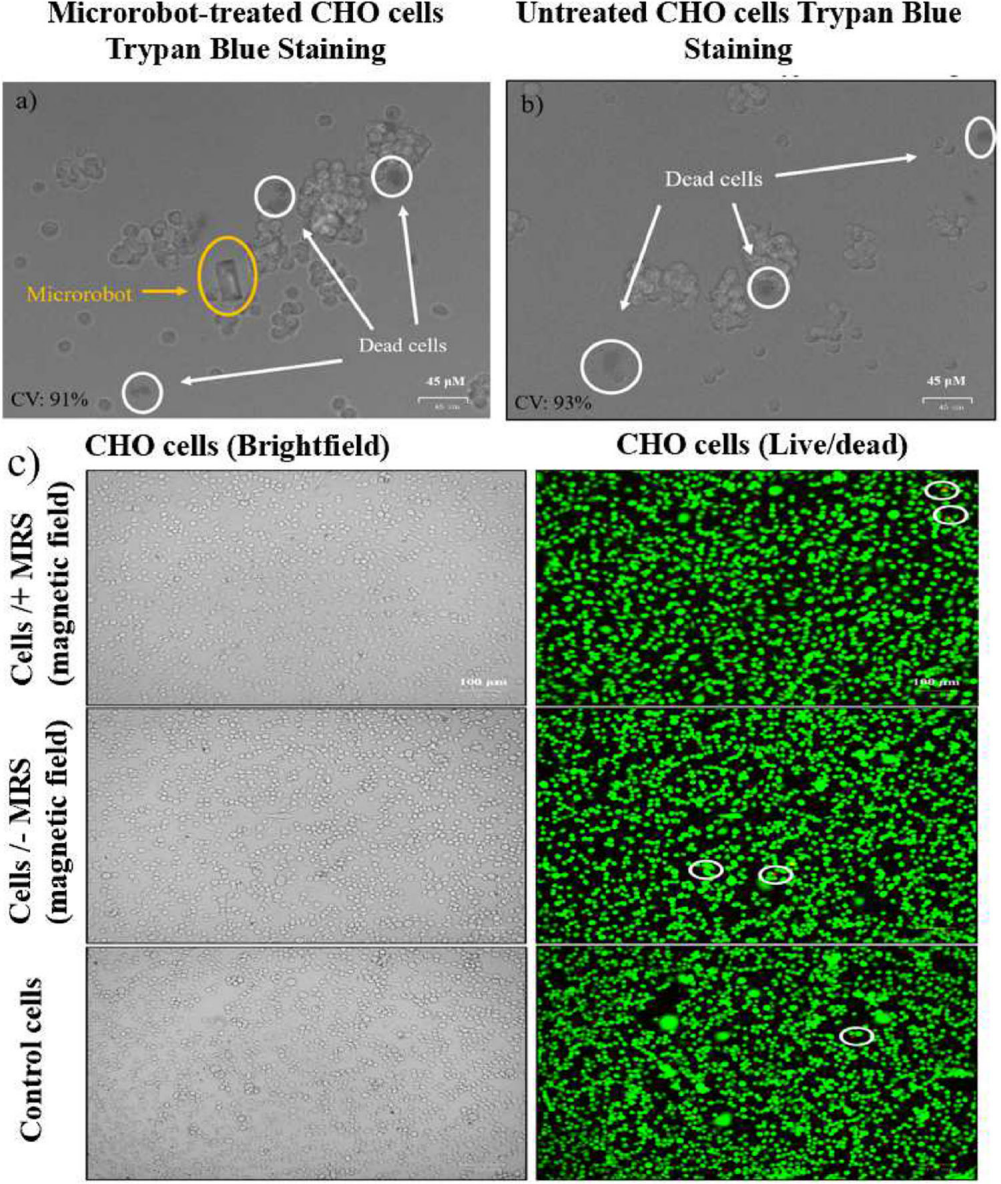
Images of trypan blue assay results with the microrobot-treated (a) and untreated (b) CHO cells after overnight incubation. Images of LIVE/DEAD Viability/Cytotoxicity Kit results in the presence of an external magnetic field and control cells (c). Dead cells are shown in white circles. CV represents cell viability

### Cell viability

Cell viability is one of the crucial factors in designing biocompatible microrobots. To assess the cytotoxicity of acoustic modular microrobots, we performed trypan blue staining and commercially available LIVE/DEAD viability tests on CHO cells, respectively. Trypan blue assay enables us to determine cell viability quickly and conveniently by selectively penetrating only dead cells’ membranes. Live cells remain unstained since they do not absorb the dye, whereas dead cells absorb the dye due to their damaged cell membranes. CHO cells were treated with the microrobots, and Fig. 8 shows the cell viability results compared to the untreated control group. Cell viability for microrobot-treated cells was 91% while it was 93% for the untreated control cells. It should be highlighted that cells that are directly in contact with the microrobots are viable after 24 hours of treatment. Trypan blue assay showed there is no significant difference in toxicity. Cytotoxicity was also checked via LIVE/DEAD viability test to evaluate the morphology of the CHO cells after microrobot treatment. Live and dead cells were stained with green and red fluorescent dye, respectively. Furthermore, we tested the direct adverse effect of the static external magnetic field on CHO cells in the presence of the microrobots. In Fig. 8, we showed the results for three groups: CHO cells with and without microrobots under a certain magnetic field, and control cells without the microrobots and magnetic field effect. According to the results of the LIVE/DEAD viability test, the number of dead cells between the three groups stays intact and nearly the same. Moreover, the cell morphology and proliferation did not alter by the treatment and the magnetic field as shown in brightfield images. CHO cells grew at a normal rate for 24 hours without showing any dramatic change in the cell morphology under the magnetic field of 4 mT after 5 minutes of exposure.

## Conclusion

In this work, we successfully demonstrated a novel multi-stimuli microrobot that can be used for making programmable modular structures. Our experimental results illustrate the assembly of different modular shapes by magnetic dipole-dipole attraction, facilitated and tuned with acoustic and magnetic fields. These microrobots provide increased flexibility, and their on-demand and on-site assembly can potentially overcome the limitations of traditional and non-modular fabrication methods. The modular microrobots were also acoustically actuated and magnetically steered, which could enable us to implement closed-loop control in the future. In addition, we demonstrated the manipulation of cells into patterns using the individual unit. Future work could include exploring the functionality and adaptability of the microrobots in complex environments, where their advantages can be fully realized. Furthermore, the capabilities of these micro assemblies in applications such as cell patterning and cargo delivery could also be investigated. Overall, these results demonstrate a novel means of modular microrobot assembly that could lead toward the creation of flexible and adjustable microrobots for micro-engineering, biomedical, and other applications.

## Supplementary Information

Below is the link to the electronic supplementary material.

Supplementary file 1 (mp4 130126 KB)

Supplementary file 2 (mp4 104648 KB)

Supplementary file 3 (mp4 45935 KB)

Supplementary file 4 (mp4 214326 KB)

## Data Availability

No datasets were generated or analysed during the current study.
